# Association of Pregnant Women’s Perinatal Depression with Sociodemographic, Anthropometric and Lifestyle Factors and Perinatal and Postnatal Outcomes: A Cross-Sectional Study

**DOI:** 10.3390/jcm13072096

**Published:** 2024-04-03

**Authors:** Constantina Jacovides, Sousana K. Papadopoulou, Eleni Pavlidou, Antonios Dakanalis, Olga Alexatou, Theofanis Vorvolakos, Eleftherios Lechouritis, Elena Papacosta, Maria Chrysafi, Maria Mitsiou, Maria Mentzelou, Rena I. Kosti, Constantinos Giaginis

**Affiliations:** 1 Department of Food Science and Nutrition, School of the Environment, University of the Aegean, 81400 Lemnos, Greece; con.jacovides@gmail.com (C.J.); elen.p.pavl@gmail.com (E.P.); rd.olga.alexatou@gmail.com (O.A.); fns18059@fns.aegean.gr (E.L.); fnsm22030@fns.aegean.gr (M.C.); maria.mentzelou@hotmail.com (M.M.); cgiaginis@aegean.gr (C.G.); 2 Department of Nutritional Sciences and Dietetics, Faculty of Health Sciences, International Hellenic University, 57001 Thessaloniki, Greece; 3 Department of Mental Health, Fondazione IRCCS San Gerardo dei Tintori, 20900 Monza, Italy; antonios.dakanalis@unimib.it; 4 School of Medicine and Surgery, University of Milano-Bicocca, 20900 Monza, Italy; 5 Department of Psychiatry, School of Health Sciences, University General Hospital of Alexandroupolis, Democritus University of Thrace, 68100 Alexandroupolis, Greece; tvorvola@med.duth.gr; 6 Department of Physical Education and Sport Sciences, School of Education and Social Sciences, Frederick University, 3080 Limassol, Cyprus; aero.pe@frederick.ac.cy; 7 Department of Physiotherapy, School of Health Sciences, International Hellenic University, 57001 Thessaloniki, Greece; mitsioumaria@gmail.com; 8 Department of Nutrition and Dietetics, School of Physical Education, Sport Science and Dietetics, University of Thessaly, 42132 Trikala, Greece; renakosti@uth.gr

**Keywords:** depression, pregnancy, sociodemographic factors, anthropometric factors, lifestyle, perinatal, preterm birth, caesarean section, gestational weight gain, breastfeeding

## Abstract

**Background:** In recent decades, the incidence of depression has gradually increased in the general population globally. Depression is also common during gestation and could result in detrimental gestational complications for both the mother and the fetus. The survey presented aimed to evaluate whether pregnant women’s perinatal depression could be associated with socio-demographic, anthropometry and lifestyle factors, and perinatal and postnatal outcomes. **Methods:** This is a cross-sectional survey conducted on 5314 pregnant women. Socio-demographic and lifestyle factors were recorded by relevant questionnaires via face-to-face interviews. Anthropometric parameters were measured by qualified personnel. Perinatal depressive symptomatology status was evaluated by Beck’s Depression Inventory (BDI-II) questionnaire. **Results:** Depressive symptoms throughout gestation were found in 35.1% of the enrolled women. Perinatal depression was significantly associated with lower educational and economic level, pre-pregnancy regular smoking and reduced levels of Mediterranean diet adherence levels, a higher prevalence of gestational diabetes and preterm birth, as well as a higher incidence of delivering by caesarean section and abnormal childbirth weight. Perinatal depression was also significantly associated with a higher prevalence of maternal postpartum depression and lower prevalence of exclusive breastfeeding practices, as well as with a higher incidence of childhood asthma. **Conclusions:** Pregnant women’s perinatal depression appears to be associated with various socio-demographic, anthropometry, and lifestyle characteristics and with a higher frequency of several adverse pregnancy complications. The present findings emphasize the importance of pregnant women’s perinatal mental health, highlighting the need to develop and apply public strategies and policies for psychological counseling and support of future mothers to minimize probable risk factors that may trigger perinatal depression. Novel well-organized, follow-up surveys of enhanced validity are highly recommended to establish more definitive conclusions.

## 1. Introduction

Depression is a very frequent mental disease which influences diverse aspects of the quality of human life [1,2]. Its main symptoms include loss of interest and appetite, anxiety and melancholy, frustration, helplessness, weakness and feelings of guilt or shame [3,4]. The World Health Organization (WHO) has reported that 25% of individuals worldwide could experience a mental disease at some time in their lives, highlighting that depression constitutes a major contributing cause to the global rise in women’s disability-adjusted life years [1,2,5]. Pregnant women undergo physiological, hormonal, and psychological changes, which could enhance the likelihood of mental health disorders [6]. Both depression and anxiety are quite common during gestation, with about 30% of pregnant women reporting some severe depressive symptoms during their gestation [7,8,9,10].

Furthermore, women of reproductive age, at a prevalence ranging from 8% to 16%, may be diagnosed with depression according to US research [11]. In contrast, some studies have found that pregnant women are protected against depression, which has been ascribed to a lower rate of suicide incidents [12,13]. A study carried out during the COVID-19 pandemic highlights that pregnant women have fewer depression symptoms compared to women who were not pregnant [14]. During the pandemic, partner support was identified as a significant predictor of decreased levels of depressive symptoms in both pregnant and non-pregnant women [14,15]. Pregnant women reported feeling more supported by their partners than non-pregnant women [14,15,16]. In periods of crisis and stress, partner support was identified as a protective factor against depression throughout the pregnancy [17,18]. Moreover, pregnant woman showed an absence of public care, which was linked to an elevated probability of perinatal depression symptomatology throughout the COVID-19 pandemic, whereas an increased perceived level of community support was related to fewer perinatal depression symptoms [19].

Previous studies have explored whether socio-demographic factors like educational level, culture, maternal age, and economic status may influence the frequency and intensity of depressive symptoms throughout gestation [20,21,22,23]. The prevalence of mental distress was shown to be enhanced during the third trimester of gestation [24]. A gradual rise in total and free cortisol plasma concentrations, which peaked in the third trimester of pregnancy, was noted [25,26]. In contrast to working women, unemployed women were more likely to suffer from emotional distress [20,21,22,23,27]. Likewise, women whose partners were employed appeared to exhibit fewer mental distress symptoms than women whose partners were unemployed [24,28,29]. The above is in line with research revealing elevated mental distress symptoms for the respondents whose family’s annual income was much lower [30]. Moreover, unemployed pregnant women were found to show an enhanced risk of preterm delivery [31,32].

There is a strong demand to investigate anthropometric characteristics like maternal body mass index (BMI) to obtain insights into the probable links between physical well-being and mental health of pregnant women. Amongst the most usual pregnancy issues, depression and obesity may trigger harmful health factors concerning both the pregnant women and their fetus [33]. Some studies have not found any significant relation between depressive symptoms and overweight or obesity in pregnant women [34]. On the other hand, multiple lines of evidence have suggested that depressed pregnant women could exhibit an elevated probability of obesity compared to those without depression [33,35]. Likewise, pregnant women with a normal body weight may be less vulnerable to suffering from depressive symptoms either during gestation or postpartum than those affected by obesity [36,37]. 

Lifestyle choices encompass dietary patterns, physical activity levels, and substance eating, and they are currently under scrutiny for their probable impacts on, by either alleviating or exacerbating, depressive symptomatology amongst pregnant women [38,39,40,41]. Certain eating habits, nutrients and foodstuffs can promote significant impacts against depression through various molecular mechanisms [42,43]. Natural products and specific nutrients such as vegetables, fruits, medicinal plants, tea, and curcumin were found to reduce oxidative stress and inflammation, enhance the nervous system’s performance, and alter biomarkers and signaling pathways related to anxiety and depressive symptoms [44,45,46,47]. These molecular mechanisms can improve monoamine neurotransmitter production and decrease the hyperactivity of the hypothalamus–pituitary–adrenal (HPA) axis [48,49,50]. Moreover, they can modify the microbiota–gut–brain axis, which decreases oxidative stress and disorders connected to inflammation [48,49,50]. The overload of HPA axis is also associated with depressive symptoms. By reducing HPA axis activation, depression was shown to be partially decreased [51,52]. In addition, while selective serotonin reuptake inhibitors (SSRIs) work pharmacologically on monoaminergic systems, they can also restore the normality of the HPA axis’ overall response, enhancing the clinical prognosis of individuals suffering from depression [53,54].

Depressed pregnant women have undesirable perinatal outcomes, such as low childbirth weight, preterm birth, and limitation of intrauterine growth [55,56]. This is vital to understand the complicated pathways through which depression during pregnancy affects fetal growth and postnatal outcomes. Several studies have also found a relationship between perinatal depression and low childbirth weight, which is linked to a higher risk of newborn morbidity and mortality [8,57]. Several surveys showed that pregnant women presenting depressive symptoms are characterized by an enhanced probability of a low childbirth weight [58,59].

The present study investigates the association of pregnant women’s perinatal depression with multiple socio-demographic, anthropometry, and lifestyle factors, as well as perinatal and postnatal outcomes. Obtaining an understanding of how these factors interact with pregnant women’s mental health can contribute towards developing effective approaches for promoting perinatal care and providing adequate support for improving the mental well-being of future mothers.

## 2. Methods

### 2.1. Study Population

**Figure 1:**
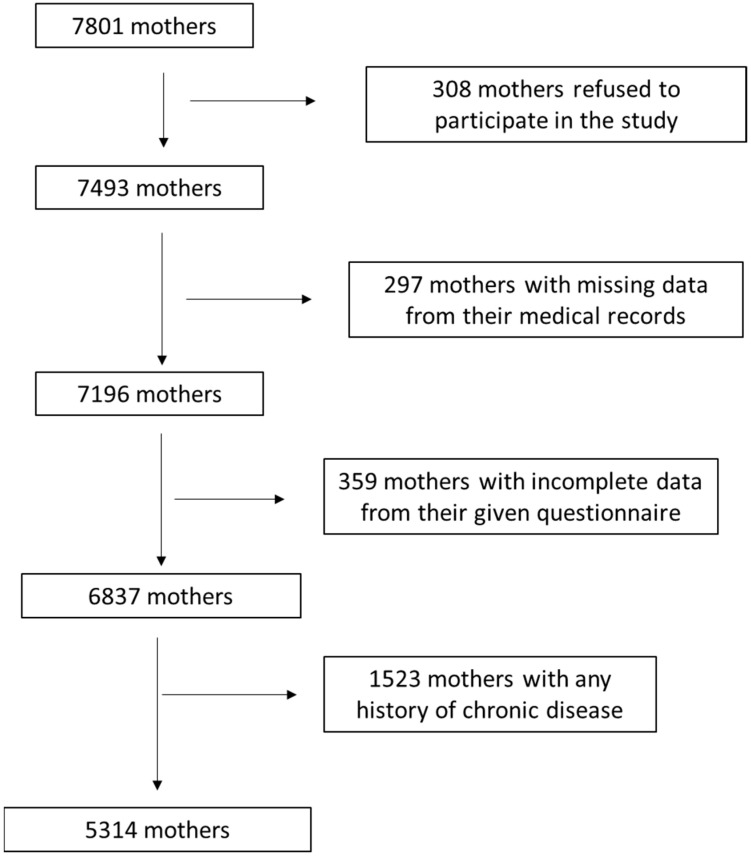
Flow chart diagram of survey population assignment.

The current survey primarily included 7801 mothers from 12 different geographic Greek regions, rural and urban (Athens, Thessaloniki, Larissa, Kavala, Alexandroupolis, Ioannina, Patra, Kalamata, Korinthos, Crete, South and North Aegean). We initially enrolled any mothers presenting a singleton birth, independently of parity. In multiparous mothers (n = 1656, 31.2%), only their latest pregnancy was considered. Enrollment to the survey was carried out between May 2016 and September 2020. Women enrollment was conducted from the beginning of the 3rd to the end of the 6th month of gestation. The enrolled mothers were followed up for nine months after the birth. Figure 1 show in detail the flow chart diagram of the participants’ assignment. By using appropriate exclusion and inclusion criteria, 5314 mothers were finally involved, leading to a final response proportion of 68.1%.

The Ethics Committee of the University of the Aegean authorized this survey (ethics approval code: No. 12/14.5.2016, approval date: 14 May 2016) in accordance with the World Health Organization (52nd WMA General Assembly, Edinburgh, Scotland, 2000). All the enrolled women data were confidential, none of the assigned women had any history of chronic disorder, including depression or any other mental disorder before pregnancy, and all participants were informed of the purposes of the present study and signed a permission document. Sample size computation was conducted utilizing PS: Power and Sample Size estimator 3.1 software. The randomization was conducted using a series of random binary numbers (i.e., 001110110, in which 0 presented enrollment and 1 not enrollment to the study). The computation of the power of our sample size showed a power of 87.9%.

### 2.2. Assessment of Sociodemographic and Anthropometric Factors

Appropriate questionnaires were applied to collect socio-demographic parameters at the time of the pregnancy’s beginning, like women age, nationality, educational level, economic status, marital and employment status, type of residence, and pre-pregnancy smoking habits, and parity of the assigned mothers was carried out through one-to-one interviews of participating pregnant women and the qualified personnel to lower the recall bias. Smoking habits concerned the pre-pregnancy period, as the vast majority (93.5%) of regular smokers interrupted smoking during their gestation. The educational level was categorized into 3 groups: (a) primary education, (b) secondary education, and (c) university studies. Family financial status was categorized according to the annual family income as low for a family annual income ≤EUR 10,000, medium for an annual income >EUR 10,000 and ≤EUR 20,000, and high for an annual income >EUR 20,000.

The body weight and height of the assigned women were measured data recovered from their medical documents to calculate Body Mass Index (BMI) at the first weeks of gestation. The women’s weight was measured by a Seca scale [Seca, Hanover, MD, USA], with no shoes, and to the closest 100 g, and height was measured by a portable stadiometer (GIMA Stadiometer 27335, Gima S.p.A., Gessate, Italy) without shoes on, to the closest 0.1 cm. The WHO guidelines were used for classifying the enrolled pregnant women as normal weight, overweight or obese at the first weeks of gestation [60,61].

### 2.3. Assessment of Lifestyle Factors

The Beck Depression Inventory (BDI-II) was applied for assessing the depressive symptomatology of the participating women during their gestation. This questionnaire comprises twenty-one classes of records and is one of the most broadly utilized psychometric examinations for evaluating the severity of depressive symptoms [62]. BDI-II contains items related to depressive symptomatology like desperation and touchiness, thoughts like shame or emotions of punishment, and physical symptoms like weakness, weight reduction, and absence of sexual attraction [62]. The BDI-II has been documented as a considerably suitable psychometric technique, representing adequate consistency and capability to classify people as depressed or not depressed, and increased simultaneous, satisfied, and structural authority. According to the existing psychometric indications, the BDI-II is identified as a cost-effective questionnaire for examining the intensity of depression symptomatology, with broad applicability for research and clinical practice worldwide. A score > 20 was applied as a cut-off to classify the enrolled pregnant women as depressed or non-depressed during gestation [62].

The Edinburgh Postnatal Depression Scale (EPDS) was applied for evaluating women’s depressive symptomatology between the 3rd and 6th month after delivery [63,64]. The EPDS contains 10 brief records. Any mother chooses one of 4 possible answers that is closest to her feelings in the last seven days [63,64]. The vast majority of the enrolled women filled the questionnaire in approximately 5 min. The responses were marked as 0, 1, 2 and 3 based on the symptoms’ severity. Objects 3, 5 to 10 were inversely counted (i.e., 3, 2, 1, and 0). The total scoring was established by joining together the scores for every of the 10 items. Enrolled women presenting a score >12 possibly felt depressed and were proposed to be supported by a qualified doctor [63,64]. This score (>12) was applied as a cut-off to classify the enrolled women as depressed or non-depressed postnatally. The scale demonstrates whether the enrolled women experienced these symptoms during the previous week, and it is helpful to use the scale once more 2 weeks afterwards. Thus, we performed the same procedure 2 weeks later to reduce possible biases. The assigned women chose the response that was nearer to how they felt in the previous 2 weeks with the additional assistance of the trained staff through one-to-one interviews. The possibility of the mother talking about her responses with others should be prevented [63,64].

Regarding MD evaluation, the well-recognized and certified MedDietScore was applied at the initial weeks of pregnancy [65,66]. This questionnaire evaluates the food incidence intake of 7 chosen foods’ categories according to MedDietScore index. Every question includes six probable responses, from 0 to 5, that depend on the level of compliance for each foods’ category. The summation of the eleven answers results in a score from 0 to 55; a greater score signifies enhanced MD compliance [65,66]. Regarding cereals, potatoes, fruits, vegetables, dairies and olive oil, the scores of 6 probable answers were related to daily intake. Concerning legumes, fish, red meat and poultry, the scores of six probable responses were related to weekly intake [65,66]. The 11th question assessed wine drinking at a per day frequency with a moderate intake (≤1 and ≤2 drinks/day for women and men, respectively; one drink = 100 mL = 12 g ethanol), being classified as the greatest score [65,66]. The enrolled women were asked to complete MedDietScore questionnaire based on their nutritional habits of the last year.

### 2.4. Assessment of Perinatal and Postnatal Outcomes

Perinatal outcomes, including gestational weight gain (GWG), childbirth body weight, preterm childbirth (<37th week), the potential presence of gestational diabetes and gestational hypertension, and the kind of childbirth (vaginal or caesarean section), were recovered from the women’ medical documents. Based on the Institute of Medicine’s (IOM) guidelines, the proposed GWG for underweight women pre-pregnancy (BMI < 18.5 kg/m^2^) was between 12.5 and 18.0 kg, for normal weight women (BMI: 18.5–24.9 kg/m^2^) between 11.6 and 16.0 kg, for overweight women (BMI: 25.0–29.9 kg/m^2^) between 7.0 and 11.5 kg, and for obese women (BMI ≥ 30.0 kg/m^2^) between 5 and 9 kg [67]. The participating women were classified according to the overhead criteria into 3 classes: women presenting a lower than recommended GWG, (b) women presenting a normal GWG, and (c) women presenting excess GWG. Childbirth body weight status was classified into three groups as low (<2500 g), normal (2500–4000 g), and high (>4000 g) [68].

Furthermore, the participating women responded if they implemented breastfeeding at all and whether they adopted exclusive breastfeeding for their newborn for at least 4 months [69,70]. To reduce recall biases, the participants answered whether they fully breastfed their newborn for at least 4 months, as, at this time, they were counselled to gradually start including pulp foods in the diet of their newborn and thus were able to more precisely remember this time point, enhancing the validity of their answers. In contrast, women implemented lactating efforts for smaller periods were not able to response with sufficient reliability concerning the accurate interval of the lactating process [69,70].

Childhood asthma was identified by expert doctors according to the International Study of Asthma and Allergies in Children and data concerning asthma-specific therapeutic approaches and healthcare utilization [71,72]. The trained staff systematically extensively explained to all assigned women how they should fill out the questionnaires in one-to-one interviews to decrease recall biases. The trained staff thoroughly explained all the questions for every questionnaire to enhance the reliability of the answers.

### 2.5. Statistical Analysis

The continuous variables following a normal distribution were treated by Student’s *t*-test. Normal distribution was tested by the Kolmogorov–Smirnov test. Chi-square was applied for categorical variables. Mean value ± Standard Deviation (SD) was used to express quantitative variables following a normal distribution. Non-normally distributed variables were treated by non-parametric analysis using the Mann–Whitney U test. The quantitative continuous variables that did not show a normal distribution were presented as a median value (Interquartile Range, IQR). The qualitative variables are presented as absolute or relative occurrences. Multivariate binary logistic regression analysis was used to assess whether women’s depressive symptomatology throughout pregnancy could independently be associated with socio-demographic, anthropometry, and lifestyle characteristics, and perinatal and postnatal outcomes, by adjusting for potential confounders. The Statistica 10.0 software, Europe, was used for the statistical analysis (Informer Technologies, Inc., Hamburg, Germany).

## 3. Results

### 3.1. Descriptive Statistics of the Study Population

Overall, 5314 mothers with a mean age of 34.7 ± 5.6 years at the beginning of their pregnancy participated in the present study. In total, 35.2% of the enrolled women showed depressive symptoms diagnosed by the BDI-II questionnaire during their gestation, while the remaining 64.9% of them did not show any depressive symptomatology. Most of the enrolled pregnant women (95.7%) were Greek. Concerning their educational level, 31.5% of the enrolled women completed primary education, 41.5% of them achieved secondary education, and 27.2% attended university. Concerning their economic status, 41.9% of the enrolled women reported a low economic status, 39.2% of them reported a medium economic status, and 18.9% reported a high economic status. In total, 70.5% of the allocated women were married and 78.8% of them were employed. In addition, 65.0% of the assigned participants lived in urban areas and the remaining 35.0% lived in rural areas. A total of 74.5% of the assigned women were not smokers pre-pregnancy, and 68.8% of them were nulliparous.

Based on the pre-pregnancy BMI classification, 72.4% of the participating women exhibited a normal BMI, 17.8% of them were categorized as overweight, and 9.8% showed obesity. According to IOM recommendations, 66.4% of the participating women showed a normal GWG, 5.4% of them showed a low GWG, and 28.2% of them had an excessive GWG. A total of 18.2% of the enrolled pregnant women had a preterm childbirth, 7.0% presented gestational diabetes, and 8.9% of them were diagnosed with gestational hypertension. Furthermore, 15.6% of the newborns exhibited a birth body weight > 4000 g, 7.1% of them presented a birth body weight < 2500 g, and the remaining 77.3% showed a normal birth body weight (2500–4000 g).

Concerning the type of delivery, 56.4% of the enrolled pregnant women had a childbirth by caesarean section and the remaining 43.6% of them had a vaginal delivery. Half (50.7%) of the assigned women exclusively breastfed their newborns for at least 4 months and 9.9% of them were diagnosed with postpartum depression. Furthermore, 5.0% of the matched children developed childhood asthma 2–5 years after delivery, and 4.4% were diagnosed with diabetes mellitus type I.

### 3.2. Association of Pregnant Women’s Perinatal Depression with Sociodemographic, Anthropometric and Lifestyle Factors

In cross-tabulation, pregnant women’s depressive symptoms were considerably related to a lower educational and financial status (Table 1, *p* = 0.0077 and *p* = 0.0007, respectively). Pregnant women with depression significantly more frequently lived in rural regions (Table 1, *p* = 0.0053). Depressed pregnant women were significantly more frequently regular smokers pre-pregnancy than non-depressed pregnant women (Table 1, *p* = 0.0381). Lower levels of MD compliance were considerably more often noted in depressed pregnant women compared to non-depressed ones (Table 1, *p* < 0.0001). Pregnant women’ age, nationality, marital and employment status, and parity did not show any significant associations or trends of correlation with depressive status during their gestation (Table 1, *p* > 0.05).

### 3.3. Association of Pregnant Women’s Perinatal Depressive Symptoms with Perinatal and Postnatal Outcomes

Both overweight and obese women prior to gestation were significantly more frequently diagnosed with depression during their pregnancy (Table 1, *p* < 0.0001). Depressed pregnant women had significantly more excessive GWG than non-depressed pregnant women (*p* < 0.0001). Perinatal depression of pregnant women was significantly associated with preterm childbirth (Table 1, *p* = 0.0008). Higher childbirth body weight status was significantly more frequently observed in depressed pregnant women compared to non-depressed pregnant women (Table 1, *p* < 0.0001).

Perinatal depression of pregnant women was significantly related to gestational diabetes (Table 1, *p* = 0.0008). In addition, pregnant women diagnosed with perinatal depressive symptoms significantly more frequently had a childbirth by caesarean section than those without perinatal depression (Table 1, *p* = 0.0001). Non-depressed pregnant women more frequently applied exclusive breastfeeding for at least 4 months compared to depressed pregnant women (Table 1, *p* < 0.0001). In addition, perinatal depression was considerably related to postpartum depression (Table 1, *p* < 0.0001). Childhood asthma at the age of 2–5 years old was more frequently observed in children of women who suffered from perinatal depression (Table 1, *p* < 0.0001). Gestational hypertension and childhood diabetes mellitus type 1 were not associated with pregnant women’s perinatal depression status (Table 1, *p* > 0.05).

### 3.4. Multivariate Binary Logistic Regression Analysis for Pregnant Women’s Perinatal Depression

In multivariate binary logistic regression analysis, pregnant women’s perinatal depression was independently related to educational and economical level, type of residence, MD compliance, pre-pregnancy overweight/obesity, GWG, premature childbirth, childbirth body weight classification, gestational diabetes, type of delivery, exclusive breastfeeding, maternal postpartum depression, and childhood asthma by adjusting for several possible confounders (Table 2, *p* < 0.05).

The enrolled participants with a lower educational level and a worse economic status exhibited a 28% and a 36% higher prevalence of presenting depressive symptomatology, respectively, throughout gestation than those with an advanced educational level and a better economic status (Table 2, *p* = 0.0276 and *p* = 0.0120, respectively). Women reporting enhanced MD compliance exhibited a more than two-fold lower frequency of perinatal depression during their pregnancy compared to women presenting reduced MD compliance (Table 2, *p* = 0.0011). Accordingly, women who were overweight or obese pre-pregnancy showed a two-fold higher likelihood of perinatal depression during their pregnancy than those with a normal, pre-pregnancy BMI status (Table 2, *p* = 0.0005). Women presenting excessive GWG showed an 86% higher prevalence of developing perinatal depression during their pregnancy compared to those presenting GWG within IOM recommendations (Table 2, *p* = 0.0003). Moreover, women who had a preterm childbirth showed a higher incidence of developing perinatal depression during their pregnancy than those who did not have a preterm childbirth (Table 2, *p* = 0.0014).

In addition, women whose children had an increased birth body weight (>4.000 g) showed a 77% higher likelihood of developing depressive symptomatology throughout their gestation than those whose children had a normal birth body weight status (Table 2, *p* = 0.0017). Women who developed gestational diabetes presented a 77% higher prevalence of perinatal depression during their gestation than women without gestational diabetes (Table 2, *p* = 0.0026).

Women presenting perinatal depressive symptomatology showed a 63% higher incidence of having a child by caesarean section than those without perinatal depressive symptomatology during their gestation (Table 2, *p* = 0.0025). Women with perinatal depressive symptoms also exhibited an almost two-fold higher likelihood of not breastfeeding their child than those without perinatal depression during their gestation (Table 2, *p* = 0.0034). Moreover, women presenting depressive symptoms throughout pregnancy showed a more than two-fold higher incidence of developing postpartum depression than those without perinatal depressive symptomatology during their gestation (Table 2, *p* = 0.0002). Lastly, women with depressive symptoms during gestation had a 72% greater incidence of delivering a child diagnosed with childhood asthma at the early stages of their life (2–5 years after delivery) than those without perinatal depressive symptomatology during their gestation (Table 2, *p* = 0.0002).

A second multivariate binary logistic regression analysis was performed including the most important factors that were independently associated with pregnant women’s perinatal depression (Table 3). In this secondary multivariate regression model, pregnant women’s Mediterranean Diet adherence, pre-pregnancy BMI, GWG, type of delivery, exclusive breastfeeding, and postpartum depression were independently associated with pregnant women’s perinatal depression (Table 3, *p* < 0.05). In contrast, maternal education level, family economic status, premature childbirth, childbirth weight status, gestational diabetes, and childhood asthma did not remain statistically significant (Table 3, *p* > 0.05).

## 4. Discussion

Recent research has provided substantial evidence into the complex relationship between pregnant women’s depression during gestation and diverse socio-demographic, anthropometry, and lifestyle factors. These findings shed light on the complicated mechanisms that can influence perinatal and postnatal outcomes. By examining these dimensions in a detailed manner, a deeper understanding of pregnant women’s well-being throughout their gestation could be achieved.

Multiple research studies have explored the relationship between pregnant women’s perinatal depression during gestation and socio-demographic characteristics. In the present study, pregnant women’s perinatal depression was associated with a lower educational level and a worse economic status, whereas women’s age, nationality, and marital and employment status were not considerably related to depression during pregnancy. In agreement with our results, several previous studies have also shown that pregnant women’s perinatal depression is related to a lower educational level and a worse economic status [73,74,75,76]. However, some studies have demonstrated conflicting results, reporting a higher or a lower prevalence of perinatal depression of pregnant women [77]. In this context, we did not find any association between perinatal depression and women’s age at the beginning of their gestation. Concerning the controversy about the participants’ age, this may be ascribed to the fact that the study of Bhat N. A. et al. included only 47 pregnant women, while our sample size is considerably higher [77].

In support of our findings, a study conducted by Smith et al. revealed a noteworthy connection between lower socioeconomic status and elevated rates of pregnant women’s perinatal depression [78]. These findings are consistent with the observations made by Weiss et al., who observed an increased occurrence of depressive symptoms in mothers with limited educational backgrounds [79]. Another cohort study conducted by Hein et al. showed that marital status, educational level, previous pregnancies, type of accommodation, and family income were risk factors of depressive symptoms during and after the gestational period [80]. Thus, it is plausible to posit that all of the above socioeconomic factors might serve as stressors, thereby influencing the onset of pregnant women’s perinatal depression. Additionally, the present study indicates that depressed pregnant women were significantly more frequently regular smoker spre-pregnancy compared to non-depressed pregnant women. In this aspect, several studies have revealed a significant association between smoking status and pregnant women’s perinatal depression risk [81,82]. Alarmingly enough, those women that recognized the danger for their fetus of persistent smoking during gestation as greater were the least probable to continue smoking throughout gestation [82].

During gestation, dietary needs are elevated due to the fetus growth and pregnant women’s metabolic requirements, especially to adequately support reproduction [83]. In this aspect, Miyake Y. et al. examined the correlation between eating consumption and depressive symptoms during pregnancy and postpartum [84]. Interestingly, this study revealed that increased fish and polyunsaturated fatty acids’ consumption was independently related to a decreased frequency of pregnant women’s perinatal depression [84]. In line with the above results, the present study found that lower levels of MD compliance were significantly more frequently observed in pregnant women diagnosed with perinatal depression. In this aspect, it is well recognized that the MD is rich in fruits, vegetables, whole grains, nuts and seeds, beans, and olive oil, moderate in fish, poultry, dairy products and wine, and presents reduced quantities of red meat and sweets [85]. Also, fish-related polyunsaturated fatty acids’ intake are the main component of MD. Moreover, in a previous study, our research group performed a cross-sectional survey on 5688 pregnant women from ten different Greek regions [86]. This study supported evidence that a higher level of MD adherence during pregnancy was related to various beneficial lifestyle characteristics that could promote mothers’ health [86]. Accordingly, elevated MD compliance has been related to a decreased probability of depressive symptomatology in several follow-up cohort surveys [87]. Moreover, MD adherence exerted substantial potential for improving depression symptomatology in individuals suffering from major or mild depression [87]. Importantly, women beginning gestation and presenting a lower quality of diet additionally displayed elevated depression symptomatology levels compared to women presenting an enhanced quality of diet when the gestation begins, and this relationship persisted unchanged during gestation [88]. On the other hand, there is no conclusive evidence so far concerning the association of Western nutritional patterns and pregnant women’s perinatal anxiety and depressive symptomatology. Nevertheless, an opposite relationship amongst the healthy nutritional patterns and pregnant women’s perinatal anxiety and depression has been indicated [89].

According to several systematic review studies, increased maternal BMI is associated with elevated levels of postpartum depression and anxiety [90]. In addition, according to Jani R. et al., an increased maternal gestational BMI was associated with a higher risk of developing perinatal depressive symptomatology [91]. Accordingly, the present study indicated that BMI > 25 kg/m^2^ and BMI > 30 kg/m^2^ prior to pregnancy were significantly associated with more frequent diagnoses of pregnant women’s depressive symptoms throughout their gestation, in agreement with the above results. Notably, according to Dachew BA et al., obesity before gestation was related to a 33% enhanced probability of perinatal depression symptomatology [92]. In agreement with the above findings, we found that overweight and obesity before gestation were related to a two-fold enhanced prevalence of pregnant women depressive symptoms during their gestation compared to that of normal-weight participants.

Furthermore, we found that depressed pregnant women considerably exhibited more excessive GWG than non-depressed pregnant women during their gestation. Accordingly, Bazzazian S. et al. demonstrated that perinatal depression and anxiety were prognostic factors of excessive GWG [93]. Based on several studies, there is also a higher risk of gestational hypertension and diabetes, as well as birth abnormalities related to elevated pre-pregnancy BMI and excessive GWG compared to the relevant recommendations [63]. In addition, another study reported that education was a sociodemographic characteristic that was substantially related to maternal BMI. Specifically, women who were affected by overweight or obesity reported a lower educational level. In this aspect, it is possible that a lower degree of education contributes to body weight increase as well as perinatal depression [94]. All the above data are in accordance with our findings except for gestational hypertension, for which we did not find any association with pregnant women’s perinatal depression. This controversy may be ascribed to the fact that our study had a significantly larger and more representative sample size compared to the previous studies.

On the other hand, the present study showed that pregnant women’s perinatal depression was associated with an elevated frequency of gestational diabetes, which is in line with other previous studies. In fact, a prospective cohort study demonstrated that pregnant women with perinatal depressive symptoms developed gestational diabetes more often [95]. Additionally, a prospective multi-part survey supported evidence that gestational diabetes diagnosis is related to an increased probability of a concurrent gestational diagnosis of perinatal depression [96]. According to a recent systematic review, there is a directional relationship between gestational diabetes and perinatal depression in pregnant women [97]. More to the point, several data support that a gestational diabetes diagnosis could result in an elevated probability of perinatal depression, and a previous event of depressive symptoms during gestation could lead to an enhanced probability of gestational diabetes [97].

High levels of depressive and anxiety symptomatology during pregnancy have been associated with obstetric problems and adverse pregnancy consequences, such as preterm birth [98]. A recent population-based, cohort survey indicated that women’s antenatal depression was related to an increased risk of pregnant women’s perinatal depressive symptoms [99]. A meta-analysis further confirmed a significant risk of preterm birth in women with perinatal depression [56]. All the above findings are in accordance with the results of the present study, where preterm childbirth was more frequently observed in depressed pregnant women. Also, in the present study, depressed pregnant women showed a higher frequency of excess childbirth body weight status. This seems to be in contrast with the study of Li et al., whose results indicated that infants of mothers experiencing perinatal depression symptomatology were more likely to experience low childbirth weight compared to infants of mothers not presenting perinatal depressive symptoms [8]. However, a more careful examination of our results reveals that the incidence of low childbirth weight was higher in depressed pregnant women compare to non-depressed pregnant women, which is in line with the findings of Li et al. [8].

Moreover, caesarean section delivery is usually associated with negative child outcomes such as allergic rhinitis, asthma, and obesity [100]. The results of the present study indicated that pregnant women with perinatal depression significantly more frequently had a childbirth with caesarean section than those without perinatal depression. Accordingly, a substantial prospective cohort study including 5209 pregnant women showed that women with perinatal depression symptomatology were considerably more likely to have a preterm birth and being subjected to caesarean section [10]. These findings are in accordance with our results concerning the association of pregnant women’s perinatal depression with the increased prevalence of both caesarean section and preterm birth. The population-based cohort study of Zhang et al. also recorded a 27.7% prevalence of perinatal depression, which is quite close to the 31.5% prevalence of pregnant women’s perinatal depression in our study population [10]. An even higher incidence (37.5%) of perinatal depression was found in the study of Khouj et al., which is also close to our findings [101]. A follow-up prospective longitudinal cohort study also demonstrated that perinatal depression was associated with an elevated risk of caesarean section [102]. The small differences on the prevalence of perinatal depression may be ascribed to the different questionnaires used to evaluate perinatal depression.

Our study supported evidence that women presenting depressive symptoms throughout pregnancy showed a more than two-fold prevalence of developing postpartum depression than those without perinatal depressive symptomatology. In accordance with our results, it was shown that depressive levels throughout gestation were directly related to postpartum depression, emphasizing the significance of testing women for depressive symptoms during gestation and postnatally [103]. A prospective survey which presented its postpartum findings of women experiencing depressive symptomatology throughout gestation was also performed [104]. The above survey supported evidence that not treated perinatal depression throughout gestation was a critical predictor of postpartum depression [104]. Notably, mothers’ mood disturbances throughout gestation were related to non-exclusive breastfeeding at 3 months [105]. In addition, this study showed that non-exclusive breastfeeding at three months was related to mothers’ depressive symptomatology postnatally at 12 months [105]. A comprehensive review reported that the currently cumulative evidence may be conclusive, taking into consideration the fact that perinatal depression symptomatology was inversely related to exclusive breastfeeding, especially from the third to sixth month postnatally [106]. The above evidence is also in agreement with our results.

The present survey has some advantages since it was conducted on a quite large representative sample of pregnant women enrolled from diverse areas of our country. Thus, the current evidence could be extrapolated to other European populations of other ethnicities. In addition, our study constitutes one of the few studies that has simultaneously examined a large number of socio-demographic, anthropometry and lifestyle factors, as well as perinatal and postnatal outcomes, in association with pregnant women’s perinatal depression. An additional strength of our survey is the fact that face-to-face interviews between the assigned women and the qualified personnel were performed to decrease recall bias. The thorough description of the instructions and the in-depth description of the questions, which were carried out through face-to-face interviews, can also reduce potential recall bias, increasing the accuracy and the validity of the participants’ responses. Also, BMI data were estimated by measured body weight and height and were not self-reported, which further reduces potential recall biases. Moreover, our survey population only included healthy women without a previous severe chronic disorder, including depression and any other mental disorder, to minimize potential comorbidities’ confounding impacts. The present survey also investigated if perinatal depressive symptoms have an independent impact after adjusting for several probable confounding effects. Finally, we used certified questionnaires such as BDI-II, EPDS, and MedDietScore, which have been proven to be very reliable and consistent by numerous international clinical studies.

It is reasonable that the present study has some limitations that should be kept in mind. Its cross-sectional design decreases the validity of establishing conclusive evidence and it suffered from the potential probability of recall bias, particularly regarding the self-reported responses, even though we carried out one-to-one interviews. Consequently, no definitive and reliable conclusions concerning causality may be obtained. Nevertheless, it should be noted that self-reported records have extensively been applied in clinical studies, indicating adequate consistency and strength to predict various outcomes. Another disadvantage of the present survey deals with the issue that BMI was applied for grouping the participating women as overweight or obese before gestation. In this aspect, body fat mass and distribution should promptly be measured and involved in future surveys to further explore and support our findings.

Moreover, we should report that there is a likelihood of the presence of unmeasured confounders, like common comorbidities of depression, including anxiety and stress, eating disorders with psychiatric backgrounds, sleep disturbances, and physical activity levels of the assigned women in spite of our systematic attempts to carry out confounding adjustments. Thus, even though we applied adjustments for various confounders, it is possible that residual confounding might affect our findings. A potential factor that may affect the results presented concerns the physical activity of the enrolled pregnant women. Although this seems to be a significant limitation of our study, the vast majority of the study participants reported low physical activity levels during their pregnancy due to their fear of miscarriage. It should be noted that in our country, pregnant women choose to be physically inactive because they have an unexplained fear of miscarriage when they become pregnant. Additionally, another disadvantage of our survey is the fact that the assigned women exhibited a considerably high age (mean age: 34.7 ± 5.6 years old). In this aspect, there is considerable evidence that gestation at an older age (age > 35 years old) may be a risk factor for adverse pregnancy outcomes [107,108]. In the last decade, gestations of older women age have become more predominant, which could be related to the elevated probability of adverse pregnancy outcomes for both the mothers and their infants [107,108].

Another disadvantage of our survey is related to the fact that in the last months of our study (April 2020), the COVID-19 pandemic spread to our country, which could result in unfavorable disruptions of daily living habits by having harmful impacts on various aspects of human well-being globally [109]. This reinforces the strong demand to perform future studies to examine whether healthy dietary patterns such as MD adherence could attenuate or even co-treat the deleterious effects of the COVID-19 pandemic in diverse aspects of people’s daily life, as well as whether the COVID-19 pandemic may negatively affect the socio-demographic, anthropometry, and lifestyle characteristics in the daily life of the general population as well as of pregnant women [110].

## 5. Conclusions

The present study showed significant relationships between pregnant women’s perinatal depression and several socio-demographic, anthropometry, and lifestyle factors, as well as perinatal and postnatal outcomes. Our findings highly emphasize the importance of supporting pregnant women’s perinatal mental health in order to minimize the development of potential adverse pregnancy outcomes. Future well-designed and well-organized prospective studies using high-quality and high-validity methodology should be performed to obtain conclusive results, also taking into consideration the short- and long-term complications of the COVID-19 pandemic. As the long-term complications of COVID-19 are increasingly manifested nowadays, dramatically affecting several aspects of mental health and the quality of life of the human population, the strong demand for designing and applying effective public strategies and policies to provide counseling to and supporting future mothers in improving their lifestyle is even more urgent.

## Figures and Tables

**Table 1:** Associations of pregnant women’s perinatal depressive symptoms with sociodemographic, anthropometric and lifestyle factors and pregnancy complications.

Characteristics (n = 5314)	Pregnant Women’s Perinatal Depression		
	No 3449 (64.9%)	Yes 1865 (35.1%)	*p*-Value
**Pregnant women’s age (mean ± SD; years)**	34.8 ± 5.8	34.6 ± 5.4	*p* = 0.2364
**Maternal nationality (n, %)**			*p* = 0.2973
Greek	3295 (95.5%)	1793 (96.1%)	
Other	154 (4.5%)	72 (3.9%)	
**Maternal education level (n, %)**			*p* = 0.0077
Primary education	1042 (30.2%)	633 (33.9%)	
Secondary education	1433 (41.6%)	763 (40.9%)	
University studies	974 (28.2%)	469 (25.2%)	
**Family economic status (n, %)**			*p* = 0.0007
Low	1434 (41.6%)	791 (42.4%)	
Medium	1314 (38.1%)	773 (51.4%)	
High	701 (20.3%)	302 (16.2%)	
**Pregnant women’s marital status (n, %)**			*p* = 0.2427
Married	2410 (69.9%)	1335 (71.6%)	
Other	1039 (30.1%)	529 (28.4%)	
**Pregnant women’s employment status (n, %)**			*p* = 0.5154
Employed	2452 (71.1%)	1310 (70.2%)	
Unemployed	997 (28.9%)	555 (29.8%)	
**Pregnant women’s type of residence**			*p* = 0.0053
Urban	2288 (66.3%)	1166 (62.5%)	
Rural	1161 (33.7%)	699 (37.5%)	
**Pre-pregnancy smoking habits (n, %)**			*p* = 0.0381
Not smokers	2601 (75.4%)	1358 (72.8%)	
Regular smokers	848 (24.6%)	507 (27.2%)	
**Pregnant women’s Mediterranean Diet adherence (n, %)**			*p* < 0.0001
Very low	739 (21.4%)	591 (31.7%)	
Low	770 (22.3%)	548 (29.4%)	
Moderate	940 (27.3%)	388 (20.8%)	
High	1000 (29.0%)	338 (18.1%)	
**Parity (n, %)**			*p* = 0.2189
Nulliparity	2394 (69.4%)	1264 (67.8%)	
Multiparity	1055 (30.6%)	601 (32.2%)	
**Maternal pre-pregnancy BMI status (n, %)**			*p* < 0.0001
Normal weight	2612 (75.7%)	1234 (66.2%)	
Overweight	553 (16.0%)	391 (21.0%)	
Obese	283 (8.3%)	239 (12.8%)	
**Gestational weight gain (n, %)**			*p* < 0.0001
Low	193 (5.6%)	92 (4.9%)	
Normal	2373 (68.8%)	1157 (62.1%)	
Excessive	883 (25.6%)	616 (33.0%)	
**Preterm birth (<37th week; n, %)**			*p* = 0.0008
No	2867 (83.1%)	1481 (79.4%)	
Yes	582 (16.9%)	384 (20.6%)	
**Childbirth weight status (n, %)**			*p* < 0.0001
<2500 g	236 (6.8%)	143 (7.7%)	
2500–4000 g	2752 (79.8%)	1355 (76.6%)	
>4000 g	461 (13.4%)	367 (19.7%)	
**Gestational diabetes (n, %)**			*p* = 0.0008
No	3242 (94.0%)	1699 (91.1%)	
Yes	207 (6.0%)	166 (8.9%)	
**Gestational hypertension (n, %)**			*p* = 0.8397
No	3140 (91.0%)	1701 (91.2%)	
Yes	309 (9.0%)	164 (8.8%)	
**Kind of delivery (n, %)**			*p* = 0.0001
Vaginal	1565 (45.4%)	736 (39.5%)	
Caesarean section	1884 (54.6%)	1129 (60.5%)	
**Exclusive breastfeeding (n, %)**			*p* < 0.0001
No	1347 (39.1%)	1271 (68.1%)	
Yes	2102 (60.9%)	594 (31.9%)	
**Postpartum depression (n, %)**			*p* < 0.0001
No	3152 (91.4%)	1636 (87.7%)	
Yes	297 (8.6%)	229 (12.3%)	
**Childhood asthma (n, %)**			*p* < 0.0001
No	3316 (96.1%)	1732 (92.9%)	
Yes	133 (3.9%)	133 (7.1%)	
**Childhood diabetes mellitus type 1 (n, %)**			*p* = 0.1850
No	3289 (95.4%)	1793 (96.1%)	
Yes	160 (4.6%)	72 (3.9%)	

**Table 2:** Multivariate binary logistic regression analysis for pregnant women’s perinatal depression.

Characteristics, (n = 5314)	Pregnant Women’s Perinatal Depression (No vs. Yes)	
	HR * (95% CI **)	*p*-Value
**Pregnant women’s age** (over/below mean)	1.02 (0.39–1.72)	*p* = 0.5833
**Maternal nationality** (Greek/Other)	0.97 (0.25–1.68)	*p* = 0.7632
**Maternal education level** (University/Primary and Secondary)	1.28 (0.87–1.71)	*p* = 0.0276
**Family economic status** (Low and Medium/High)	1.36 (1.03–1.68)	*p* = 0.0120
**Pregnant women’s marital status** (Married/Other)	0.96 (0.25–1.69)	*p* = 0.6874
**Pregnant women’s employment status** (Employed/Unemployed)	0.95 (0.21–1.75)	*p* = 0.7097
**Pregnant women’s type of residence** (Rural/Urban)	1.31 (0.98–1.67)	*p* = 0.0783
**Pre-pregnancy smoking habits** (Not Smokers/Regular Smokers)	1.12 (0.78–1.57)	*p* = 0.1843
**Pregnant women’s Mediterranean Diet adherence** (Moderate + High/Very Low + Low)	2.18 (1.97–2.40)	*p* = 0.0011
**Parity** (Nulliparity/Multiparity)	0.97 (0.28–1.73)	*p* = 0.4509
**Maternal pre-pregnancy BMI status** (Normal/Overweight + Obese)	2.05 (1.87–2.24)	*p* = 0.0005
**Gestational weight gain** (Low + Normal/Excessive)	1.86 (1.65–2.07)	*p* = 0.0003
**Premature childbirth** (No/Yes)	1.48 (1.21–1.74)	*p* = 0.0014
**Childbirth weight status** (<4000 g/>4000 g)	1.77 (1.56–1.98)	*p* = 0.0017
**Gestational diabetes** (No/Yes)	1.55 (1.29–1.82)	*p* = 0.0026
**Gestational hypertension** (No/Yes)	0.96 (0.22–1.87)	*p* = 0.9372
**Type of delivery** (Vaginal/Caesarean section)	1.63 (1.32–1.95)	*p* = 0.0025
**Exclusive breastfeeding (No/Yes)**	1.95 (1.73–2.18)	*p* = 0.0034
**Postpartum depression (No/Yes)**	2.48 (2.31–2.62)	*p* = 0.0002
**Childhood asthma (No/Yes)**	1.72 (1.49–1.98)	*p* = 0.0021
**Childhood diabetes mellitus type 1 (No/Yes)**	0.97 (0.36–1.59)	*p* = 0.3875

* Hazard Ratio: HR, ** CI: Confidence Interval.

**Table 3:** Multivariate binary logistic regression analysis including the most important factors that may be independently associated with pregnant women’s perinatal depression.

Characteristics, (n = 5314)	Pregnant Women’s Perinatal Depression (No vs. Yes)	
	HR * (95% CI **)	*p*-Value
**Maternal education level** (University/Primary and Secondary)	1.17 (0.72–1.94)	*p* = 0.0835
**Family economic status** (Low and Medium/High)	1.22 (0.81–1.92)	*p* = 0.1453
**Pregnant women’s Mediterranean Diet adherence** (Moderate + High/Very Low + Low)	2.24 (1.99–2.43)	*p* = 0.0018
**Maternal pre-pregnancy BMI status** (Normal/Overweight + Obese)	2.09 (1.82–2.31)	*p* = 0.0013
**Gestational weight gain** (Low + Normal/Excessive)	1.88 (1.63–2.11)	*p* = 0.0008
**Premature childbirth** (No/Yes)	1.43 (1.01–1.98)	*p* = 0.1022
**Childbirth weight status** (<4000 g/>4000 g)	1.75 (1.26–2.23)	*p* = 0.0746
**Gestational diabetes** (No/Yes)	1.51 (1.12–1.98)	*p* = 0.1108
**Type of delivery** (Vaginal/Caesarean section)	1.65 (1.28–2.05)	*p* = 0.0291
**Exclusive breastfeeding (No/Yes)**	1.98 (1.65–2.29)	*p* = 0.0201
**Postpartum depression (No/Yes)**	2.55 (2.21–2.62)	*p* = 0.0015
**Childhood asthma (No/Yes)**	1.61 (1.30–2.17)	*p* = 0.0976

* Hazard Ratio: HR, ** CI: Confidence Interval.

## Data Availability

The data of the present study are available upon request from the corresponding author due to private policy.
